# Assessment of Cortical Auditory Function Using Electrophysiological and Neuropsychological Measurements in Children with Bone-Anchored Hearing Aids

**DOI:** 10.25122/jml-2019-0097

**Published:** 2020

**Authors:** Cristina Pantelemon, Violeta Necula, Livia Livint Popa, Steluta Palade, Stefan Strilciuc, Dafin Fior Muresanu

**Affiliations:** 1. Department of Neurosciences, “Iuliu Hatieganu” University of Medicine and Pharmacy, Cluj-Napoca, Romania; 2. “RoNeuro” Institute for Neurological Research and Diagnostic, Cluj-Napoca, Romania; 3. Department of ENT, “Iuliu Hatieganu” University of Medicine and Pharmacy, Cluj-Napoca, Romania; 4. Department of Pediatric Neurology, Children's Emergency Hospital Cluj-Napoca, Cluj-Napoca, Romania; 5. Department of Public Health, Babes-Bolyai University, Cluj-Napoca, Romania

**Keywords:** cortical auditory function, hearing loss, BAHA System, cortical auditory evoked potentials (CAEPs), children

## Abstract

Children suffering from conductive or mixed hearing loss may benefit from a bone-anchored hearing aid system (BAHA Attract implantable prosthesis). After audiological rehabilitation, different aspects of development are improving. The objective of this case report is to propose a comprehensive framework for monitoring cortical auditory function after implantation of a bone-anchored hearing aid system by using electrophysiological and neuropsychological measurements.

We present the case of a seven-year-old boy with a congenital hearing loss due to a plurimalformative syndrome, including outer and middle ear malformation. After the diagnosis of hearing loss and the audiological rehabilitation with a BAHA Attract implantable prosthesis, the cortical auditory evoked potentials were recorded. We performed a neuropsychological evaluation using the Wechsler Intelligence Scale for Children – Fourth Edition, which was applied according to a standard procedure.

The P1 latency was delayed according to the age (an objective biomarker for quantifying cortical auditory function). The neuropsychological evaluation revealed that the child's working memory and verbal reasoning abilities were in the borderline range comparing with his nonverbal reasoning abilities and processing abilities, which were in the average and below-average range, respectively.

Cortical auditory evoked potentials, along with neuropsychological evaluation, could be an essential tool for monitoring cortical auditory function in children with hearing loss after a bone-anchored hearing aid implantation.

## Introduction

Hearing loss, especially in its most severe forms, is associated with a developmental risk in the area of speech and language skills [1] and also non-verbal cognitive processes [2]. For this reason, the rehabilitation process must start as early as possible. Cortical auditory evoked potentials (CAEPs) can be used for assessing hearing sensitivity, central auditory processing, and the neural encoding of speech sound [3]. In children with normal hearing, the CAEPs morphology is governed by a positive peak known as the P1 component. In small children, it has a latency of approximately 200-300 ms [4]. As the auditory cortex develops, the P1 CAEPs response decreases rapidly in infancy, and continue to do so gradually until adulthood when it reaches a latency of approximately 50-70 ms [5]. The gradual decrease in P1 wave latency reflects an increase in the efficiency of transmitting sound along the auditory pathway in the auditory cortex [4, 6, 7] and can be used as an objective parameter to assess the efficacy of hearing aid use in children with hearing loss [4].

When cortical regions do not receive the appropriate sensory stimulus (e.g., auditory cortex in hearing loss), they are more likely to be recruited by other sensory senses (e.g., vision), resulting in a cross-modal reorganization [4]. Sharma et al. showed evidence of somato-sensitive activation of the auditory cortex in patients with long-term hearing loss [8, 9]. Auditory deprivation determines abnormally delayed latencies and morphological changes to the P1 waveform, and consequently, it affects speech, language, and cognitive processes [3]. Approximately 30-40% of children with hearing loss have associated disabilities: psychomotor developmental delay, visual impairment, cognitive impairment, language disorder, brain structural changes, and psychiatric disorders [10].

In children with hearing loss and associated disabilities, an aspect that should be considered is the benefit obtained from the audiological intervention [10, 11].

Patients suffering from conductive or mixed hearing loss may benefit from implantable hearing devices. The bone-anchored hearing aid system (BAHA System) is an implanted device that uses bone conduction in order to stimulate the cochlea, bypassing the outer and middle ear. Sound signals are converted into electrical impulses and transmitted to the brain. In individuals with congenital ear malformations (who are unable to use conventional hearing aids on air conduction), the BAHA System represents a good rehabilitation alternative [12, 13].

After the audiological intervention, different aspects of development are improving. The neuropsychological evaluation needs to establish a comprehensive framework for monitoring different dynamics of development beyond typical language skills.

We report a case in which we assess the cortical auditory function by combining electrophysiological and neuropsychological measurements. The audiological intervention was performed with the BAHA Attract implantable prosthesis.

### Subject

We present the case of a 7-year-old boy who was diagnosed with plurimalformative syndrome at birth, presenting clinical features that could not be included in a specific syndrome. He was born at term after an uneventful pregnancy (mother followed isotretinoin therapy in the first two weeks of pregnancy), spontaneous delivery, birth weight = 3380 grams, APGAR score 10. A craniofacial dysmorphism was discovered: agenesis of the right auricle, severe dysplasia of the left auricle, bilateral external auditory canal atresia, microretrognathia, broad nose implantation – hypertelorism, mongoloid palpebral fissures, severe hypoplasia of the right and moderate hypoplasia of the left upper jaw, palpebral fissures asymmetry (left < right), lagophthalmia (2-3 mm bilaterally), nasolabial asymmetry (more significant on the right), oculomotricity deficiency (right eye abduction paralysis), clinodactyly of the fifth finger, bilateral flat foot.

Temporal bone computed tomography scan revealed dysplastic ossicles, unidentifiable stapes, and oval window, hypopneumatization of mastoid cells, hypoplasia of the tympanic cavity, without changes in the internal ear. The contour of the facial nerve in the mastoid segment was not clearly defined.

### Experimental setup and procedure

Following the investigations, the diagnosis of moderate conductive hearing loss in the left ear and severe mixed hearing loss in the right ear was established. On auditory brainstem response, the V wave was present at 70 dB HL in the right ear and 80 dB HL in the left ear. From 1 month and a half, hearing rehabilitation was made with bilateral BAHA Softband. Audiological reevaluation at age 4 revealed the following audiological profile: Auditory brainstem responses (ABR) on bone conduction – V-wave present at 10 and 20 dB HL in the left ear (LE) and at 20 and 30 dB HL in the right ear (RE), auditory steady-state response (ASSR) – between 60 and 70 dB HL in the LE and 80 and 90 dB HL in the RE.

SmartEP Intelligent Hearing System (IHS) equipment (Miami, Florida, USA) was used for ABR and ASSR. Air conduction was tested with headphones, for atretic ears, and with a B-71 transducer for bone conduction, held by one finger on the mastoid.

At 6 years and 8 months, a BAHA Attract was implanted on the left ear and three months later on the right ear. At 7 years and one month, we recorded the cortical auditory evoked potentials on the aided condition to quantify the maturation of the central auditory pathways.

The P1 CAEP records were obtained after 90 minutes of testing in a soundproofed room. The patient was placed on a chair, and he watched cartoons without sound during the procedure. The patient was using a bilateral BAHA Attract implantable prosthesis. The electrodes were placed according to the norms of the International Electrode System 10-20: the active Cz electrode was connected to the positive input of the amplifier, the reference electrode was positioned on the mastoid of the ear, and the ground electrode was placed at Fpz. To minimize the ocular artifacts, a supraorbital electrode was used, paired with an infraorbital reference electrode placed ipsilaterally. The level of impedance of the electrodes was maintained between 1-3 kOhms. A calibrated loudspeaker placed at 1 m distance in 0° angle emitted a speech stimulus, the “ba” syllable, at 70 dB nHL intensity. The stimulus rate was 1.10/s, duration 114875 µsec, for 512 sweeps, artifact rejection criterion at ± 100 µV. The stimulus CAEPSs recorded in response was analyzed by a SmartEP USB software from the Intelligent Hearing System.

The check-up audiogram performed at 7 years and 4 months of age showed hearing thresholds between 20 and 30 dB HL for the RE.

Neuropsychological functioning was performed at the age of 7 years and one month, by a clinical neuropsychologist using the Wechsler Intelligence Scale for Children – Fourth Edition (WISC-IV), adapted in Romania in 2012 [14].

The assessment was based on oral/acoustic modality, which refers to the use of spoken language with auditory amplification (Baha Attract System), without visual instructions; the administration was possible without modifications, and all the tests were applied according to a standard procedure. The patient was administered ten subtests of the Wechsler Intelligence Scale for Children – Fourth Edition (WISC IV). WISC-IV measures general intelligence and specific indexes, including verbal comprehension, perceptual reasoning, working memory, and processing speed.

Verbal Comprehension Index (VCI) measures verbal concept formation. The subtests included are Similarities, Vocabulary and Comprehension.

Perceptual Reasoning Index (PRI) measures non-verbal and fluid reasoning. The subtests included are Block Design, Picture Concepts, and Matrix Reasoning.

Working Memory Index (WMI) measures working memory. The subtests included are Digit Span and Letter-Number Sequencing.

Processing Speed Index (PSI) measures the speed of information processing. The subtests included are Coding and Symbol Search.

**Figure 1:**
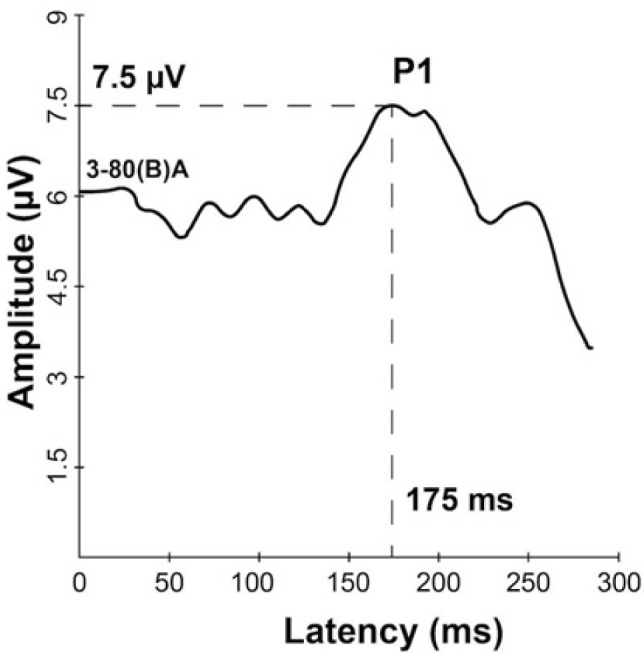
Grand average CAEP response after hearing aid intervention.

The P1 wave was observed with normal morphology and a delayed latency (175 ms) for this age (Figure 1). No artifacts caused by BAHA were observed, as described by Rahne et al. [15]. This latency value indicates that there is a delay in the maturation of the central auditory pathways.

## Discussion

The objective of this study was to assess the cortical auditory function in a child with a BAHA implantation using electrophysiological and neuropsychological measurements.

The P1 biomarker represents an objective method of assessing the development of the central auditory pathway in children with hearing loss [10]. We observed that the P1 wave has a normal morphology, but latency is delayed according to the age. Sharma et al. described in their study the values of P1 latency obtained in a group of normal children between 8 and 12 years old and found out that the average value was 81 ms for children aged seven years old [16].

Cortical auditory evoked potentials originate from the cortical-thalamic projections, the latter being involved in working memory [17].

Delayed latency of the P1 component can be correlated with working memory. The cortex development and cognitive abilities are interdependent from one another [18].

In our patient's case, there were no CAEPs measurements before the BAHA implantation; therefore, we could not compare the latencies before and after the results.

The patient's general cognitive ability is below the average range of intellectual functioning, as measured by the Full-Scale Intelligence Quotient (FSIQ). His overall thinking and reasoning abilities exceed only approximately 12% of children having his age (FSIQ=82, 95% confidence interval=78-86) (Table 1).

**Table 1:** Qualitative Descriptions of IQ Scores (Wescher, 2012)

Score	Classification	Percentage included in the theoretical normal curve
130 and above	Very Superior	2.2
120-129	Superior	6.7
110-119	High Average	16.1
90-109	Average	50.0
80-89	Low Average	16.1
70-79	Borderline	6.7
60 and below	Extremely low	2.2

**Table 2:** WISC IV Scores - Summary

WISC –IV Composite	Score	Classification
Verbal Comprehension Index (VCI)	78	Bordeline
Perceptual Reasoning Index (PRI)	89	Below Average
Working Memory Index (WMI)	77	Bordeline
Processing Speed Index (PSI)	100	Average
Full Scale IQ (FSIQ)	82	Below Average

**Table 3:** WISC IV Subtests Scores - Summary

**A. VCI (Verbal Comprehension Index)**	**Score**	**m**	**SD**
1. Comprehension	9	10	3
2. Similarities	3	10	3
3. Vocabulary	6	10	3
**B. PRI (Perceptual Reasoning Index)**			
1. Block design	9	10	3
2. Picture concept	9	10	3
3. Matrix reasoning	7	10	3
**C. WMI (Working Memory Index)**			
1. Digit span	8	10	3
2. Letter-Number sequencing	4	10	3
**D. PSI (Processing Speed Index)**			
1. Coding-Digit Symbol	12	10	3
2. Symbol search	8	10	3

His ability to think with words is lower than his ability to reason without the use of words. His verbal reasoning abilities, as measured by the Verbal Comprehension Index (VCI), are within the borderline range and below those of approximately 93% of his peers (VCI = 78; seventh percentile, 95% confidence interval = 73 – 86) (Table 1). The Verbal Comprehension Index is designed to measure verbal reasoning and concept formation. The patient's performance on the subtests that contribute to the VCI varies somehow, suggesting that his abilities in this domain are less equally developed. This can influence the child's later writing and reading skills. Early diagnosis of hearing loss followed by appropriate intervention allows for linguistic and literacy skills to be maximized, similar to those of normal-hearing children [19].

The patient's nonverbal reasoning abilities as measured by the Perceptual Reasoning Index (PRI) are below the average range and below those of approximately 67% of his peers (PRI = 89; 23rd percentile, 95% confidence interval = 83 – 97) (Table 1). The PRI is designed to measure nonverbal concept formation, visual perception and organization, simultaneous processing, visual-motor coordination, learning, and the ability to separate figure and ground in visual stimuli. The patient's performance on the subtests that contribute to the PRI is all within the average range and below average, suggesting that his abilities in this domain are similarly developed.

The subject's working memory abilities, as measured by the Working Memory Index (WMI), are within the borderline range, below those of 93% of his peers (WMI = 77; seventh percentile, 95% confidence interval = 71–86) (Table 1). The patient's abilities to maintain attention, concentrate, and exert mental control are weaknesses relative to his perceptual reasoning abilities. Mental control is the ability to attend and hold information in short-term memory while performing some operation or manipulation with it. The patient's difficulty in the working memory domain is evidence of weak mental control. Pisoni et al. (2011) showed that children with hearing loss have lower scores in working memory assessment tests compared to children with normal hearing [20]. Lo et al. demonstrated in a study that memory capacity influences the acquisition of language in hearing-impaired children. In hearing-impaired children with a long interval of working memory, the results obtained were similar to those of children with normal hearing for both receptive and expressive language. Instead, children with hearing loss and a short working memory range had lower scores in assessing expressive and responsive language [21].

The patient's speed of processing abilities as measured by the Processing Speed Index (PSI) is within the average range and above those of approximately 50% of his peers (PSI = 100; 50^th^ percentile, 95% CI = 94-106) (Table 1). Processing visual material quickly is an ability that the patient performs better as opposed to his verbal reasoning ability. Processing speed is an indication of the rapidity with which the patient can mentally process simple or routine information without making errors. The performance of this task may be influenced by visual discrimination and visual-motor coordination. The patient achieved his best performance among the processing speed tasks on the Coding-Digit Symbol subtest (Scaled Score = 12) and the lowest score on the Similarities subtest (Scaled Score = 3) and Letter-Number Sequencing (Scaled Score = 4) (Tables 2, 3). His performance across these areas differs significantly, suggesting that these are the areas of most pronounced strength and weakness, respectively, in the patient's profile of verbal reasoning abilities and working memory.

Wolff and Thatcher et al. have shown using electrophysiological studies that in deaf children, there is a delay in maturation of the left frontotemporal and bilateral frontal regions [22]. Lack of hearing input secondary to deafness leads to reduced frontal hearing connections [23], affecting the neural organization of the frontal and prefrontal cortex [24]. The delay in cortical maturation at this level may have effects on the motor skills sequence in language development as well as other aspects of cognitive function [20].

Daza et al. have shown that vocabulary knowledge and non-verbal cognitive processes such as selective attention, visual-spatial memory, abstract reasoning, and sequential processing are predictive factors for reading comprehension in deaf children [25].

The purpose of this case presentation is to come in support of the need and importance of a comprehensive assessment of developmental evolution in children with hearing loss after a BAHA implantation. The neuropsychological evaluation needs to establish a comprehensive framework for monitoring the child's performances in each developmental area. With this standardized and normed tests for the Romanian population, researchers will be able to investigate outcomes and provide accurate information to offer guidance towards the best intervention in clinical practice. However, more extensive studies are necessary to confirm the findings presented here.

## Conclusion

Cortical evoked potentials, along with neuropsychological evaluation, could be an essential tool for cortical auditory functionality and a useful clinical instrument for quantifying the outcomes of auditory rehabilitation in children with hearing loss after a BAHA implantation.

## Conflict of Interest

The authors confirm that there are no conflicts of interest.
